# Reactivation of Herpes Zoster in a Young Patient With Multiple Sclerosis Under Dimethyl Fumarate Treatment and Normal Lymphocyte Subsets Count: A Case Report

**DOI:** 10.7759/cureus.51412

**Published:** 2023-12-31

**Authors:** Razan Z AlShammari, Fatimah A AlOqayli, Saleh K Alnafeesy, Ibtisam Al Thubaiti

**Affiliations:** 1 Department of Family Medicine, Armed Forces Hospitals, Dhahran, SAU; 2 College of Medicine, Imam Abdulrahman Bin Faisal University, Dammam, SAU; 3 Department of Neurology, King Fahd Military Medical Complex, Dhahran, SAU

**Keywords:** autoimmune disease, lymphocytopenia, chickenpox, zoster, varicella, shingles

## Abstract

Herpes zoster (HZ) infection results from the reactivation of the varicella-zoster virus (VZV), which remains dormant in the dorsal root ganglia after an initial chickenpox infection. Although HZ appears more common in people with multiple sclerosis (MS) than expected in the general population, few studies have investigated this association, particularly with a normal absolute lymphocyte count (ALC). Additionally, no reported cases have discussed the clinical presentation of such patients. This report describes the case of a 26-year-old female with a known history of relapsing-remitting MS on dimethyl fumarate (DMF) treatment. She presented with a history of painful erythematous blisters, diagnosed as acute HZ infection with a normal ALC. This case provides evidence that warrants further research and attention to the management of patients with MS receiving DMF, particularly regarding infectious risks. It highlights the importance of pharmacovigilance and the potential benefits of VZV and HZ immunization in DMF recipients.

## Introduction

Varicella-zoster virus (VZV), a ubiquitous herpes virus, primarily targets the nervous system. Its initial infection, chickenpox, is a highly contagious childhood illness. After initial infection, the virus remains dormant in specific nerve ganglia within the spinal cord or cranial nerves. Reactivation of VZV manifests as herpes zoster (HZ) or shingles, characterized by a painful, papulovesicular rash along affected dermatomes [1,2]. This rash, typically lasting for two to four weeks, often comes with pain, itching, tingling, or numbness [3].

Globally, HZ afflicts approximately three to four individuals per 1,000 annually, with most cases occurring in people over 60 years [4]. Notably, HZ incidence has increased in recent decades across various regions [5]. This increase is influenced by factors impacting host immunity, such as advanced age, autoimmune diseases, cellular immune dysfunction, and past chemotherapy or steroid treatment [6].

Multiple sclerosis (MS), a chronic inflammatory demyelinating disease of the central nervous system, is driven by immune-mediated processes [7]. While the precise cause of MS remains elusive, research suggests that certain viruses might trigger autoimmune reactions in genetically susceptible individuals, potentially leading to MS development. Herpes simplex virus (HSV), VZV, human herpes virus-6, cytomegalovirus, and notably Epstein-Barr virus from the *Herpesviridae *family are some of the viruses implicated in MS [8]. Studies have shown that patients with MS have a higher rate of VZV seropositivity compared to the general population, putting them at increased risk of HZ [9-11].

These results hold significance, given the current landscape of MS treatment. While novel immunosuppressive and immunomodulatory therapies are available, T cell-mediated immunity, not merely antibody presence, provides long-lasting protection against VZV. Therapies targeting T cells, therefore, may inadvertently dampen immune responses to the virus [12]. Dimethyl fumarate (DMF) is a medication approved globally since 2013 for treating relapsing-remitting MS (RRMS) [13]. Studies have established its effectiveness and safety profile [14]. However, DMF has been shown to reduce leukocyte and lymphocyte counts, which usually normalize over time. Additionally, it noticeably decreases CD19+ B cells, CD3+ cells, and CD8+ T cells [15-20], potentially increasing the risk of shingles development while on DMF [21].

Despite the apparent increased risk of HZ in patients with MS compared to the general population [11], few studies have explored the association specifically with absolute lymphocyte count (ALC). Moreover, no reported cases have discussed HZ occurring with normal ALC. This article presents the natural history of HZ infection in a young woman with RRMS on DMF treatment who had a normal ALC.

## Case presentation

**Figure 1:**
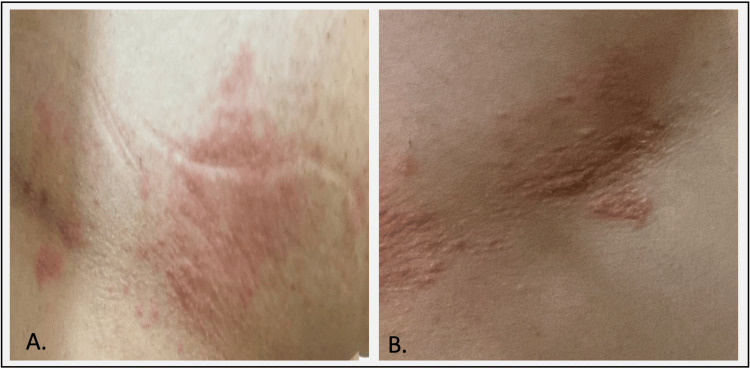
Clinical presentation of herpes zoster reactivation in a 26-year-old female with relapsing-remitting multiple sclerosis (A) Grouped erythematous maculopapular rash at the right-sided anterolateral T4 and T5 dermatomes. (B) The rash progresses to develop clusters of clear vesicles.

A 26-year-old Saudi woman with a history of RRMS presented with painful, erythematous blisters four years into treatment with DMF. She contracted chickenpox at the age of 17 and received an RRMS diagnosis at the age of 21. Clinically and radiologically stable on DMF for four years, she developed a papulovesicular rash on an erythematous base on the right side of her upper torso, specifically affecting the T4 and T5 dermatomes (Figure 1). This rash, accompanied by her skin sensitivity, tingling, and burning pain, prompted a diagnosis of HZ infection.

Treatment with oral valaciclovir 1000 mg three times daily for seven days led to rapid resolution. Within 14 days, all blisters rusted over, and the rash gradually faded, leaving moderate scarring. Laboratory tests, including white blood cell (WBC), and ALC remained within normal limits (Table 1). The patient made a full recovery within a month, experiencing only intermittent mild itchiness. DMF therapy was resumed following the complete resolution of the HZ infection, and she planned to receive zoster vaccination one year after this episode.

**Table 1:** Lymphocyte immunophenotyping at the time of HZ diagnosis WBCs: White blood cells; ALC: Absolute lymphocyte count; CD: Cluster of differentiation; NK: Natural killer; VZV: Varicella zoster virus; Ig: Immunoglobulin; Ab: Antibody; HZ: Herpes zoster.

Blood investigation test	Value	Reference range
WBCs	8.5	4.0-11.0 (10^3^/uL)
ALC	1651.7	990-3150
T cells (%)	67.5	55-84
T cells absolute	1114.9	690-2540
B cells (%)	18.3	6-25
B cells absolute	300.7	90-660
NK cells (%)	13.3	5-27
NK cells absolute	219.3	90-590
CD4+ T Cells (%)	52	31-60
CD4+ T cells absolute	861.5	410-1590
CD8+ T cells (%)	15	13-41
CD8+ T cells absolute	249	190-1140
CD4:CD8 ratio	3.5	≥ 1
(VZV) titers (VZV) IgM Ab (VZV) IgG Ab	Negative (<0.90); Positive (>4000) mlU/mL	

## Discussion

DMF use in patients with MS is associated with a decrease in ALC, but this decline generally stabilizes over time, with most maintaining ALCs above the lower limit of normal [22]. However, a small percentage [22] may experience persistently low ALCs (<500 mm3) for six months or longer, increasing the risk of severe, prolonged lymphopenia [22]. Interestingly, DMF efficacy appears independent of lymphopenia status [22]. Nevertheless, monitoring ALCs in DMF-treated patients remains crucial for identifying those at risk of developing prolonged moderate-to-severe lymphopenia [23].

This case of a young patient with RRMS on DMF with normal ALCs who developed HZ infection raises important considerations. While DMF generally does not significantly increase the risk of serious infections and is known to reduce CD8+ T cells [24], exceptions exist that challenge this notion [25]. For example, a case of VZV reactivation in a patient with MS on DMF with normal to grade 1 lymphopenia has been reported [26]. Another case demonstrated a significant decrease in CD8+ and CD4+ T lymphocytes in a patient receiving DMF, suggesting a potential link between DMF-induced lymphopenia and VZV reactivation with disseminated zoster [27]. Furthermore, a recent study found elevated CD4+/CD8+ ratios in patients with MS on DMF who developed HZ [28]. Additionally, a case of progressive multifocal leukoencephalopathy in DMF-treated patients without severe lymphopenia underscores the need for vigilance [29] as does the case of HSV encephalitis in a lymphopenic DMF-treated patient with MS, highlighting the potential implications of DMF-related lymphopenia for viral immunity [30].

Regarding HZ prevention, the Advisory Committee on Immunization Practices in the United States and the Centers for Disease Control and Prevention recommend the recombinant zoster vaccine (RZV, Shingrix) for immunocompromised adults aged 19 and older [31,32]. This vaccine, containing recombinant glycoprotein E and an adjuvant, has demonstrated moderate to high efficacy and a favorable safety profile [31]. It significantly reduces HZ risk by over 90% and is also recommended for immunocompetent adults aged 50 years and older [33]. While no studies have specifically investigated Shingrix in patients with MS, healthcare providers should consider its use for patients with suspected MS to prevent HZ and related complications.

## Conclusions

These cases compellingly highlight the need for further research and attention in managing patients with MS receiving DMF, particularly regarding infections. Carefully weighing the potential risks and benefits of DMF is crucial, and healthcare professionals should remain vigilant in monitoring ALCs for DMF recipients. This uncommon case, despite its rarity, underscores pharmacovigilance concerns and the potential benefits of both VZV and HZ vaccination in DMF patients.
